# Surfactant-Mediated Assembly of Precision-Size Liposomes

**DOI:** 10.1021/acs.chemmater.4c01127

**Published:** 2024-07-25

**Authors:** Ivan S. Pires, Jack R. Suggs, Isabella S. Carlo, DongSoo Yun, Paula T. Hammond, Darrell J. Irvine

**Affiliations:** † Koch Institute for Integrative Cancer Research, Massachusetts Institute of Technology, 500 Main Street, Cambridge, Massachusetts 02139, United States; ‡ Department of Chemical Engineering, Massachusetts Institute of Technology, 21 Ames Street, Cambridge, Massachusetts 02139, United States; § Department of Biological Engineering, Massachusetts Institute of Technology, 25 Ames Street, Cambridge, Massachusetts 02139, United States; ∥ Department of Materials Science and Engineering, Massachusetts Institute of Technology, Cambridge, Massachusetts 02139, United States; ⊥ Ragon Institute of Massachusetts General Hospital, Massachusetts Institute of Technology and Harvard University, Cambridge, Massachusetts 02139, United States; # Howard Hughes Medical Institute, Chevy Chase, Maryland 20815, United States

## Abstract

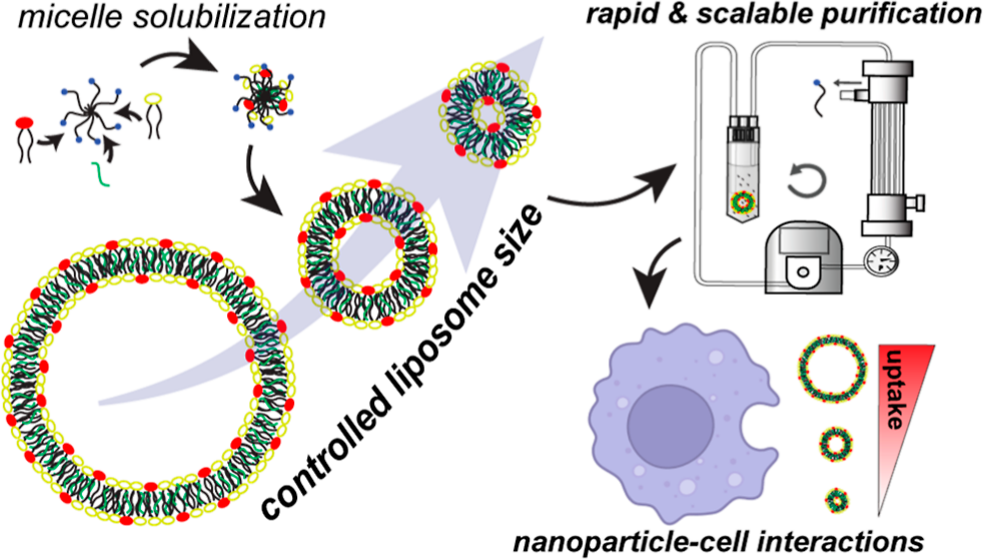

Liposomes can greatly improve the pharmacokinetics of therapeutic agents due to their ability to encapsulate drugs and accumulate in target tissues. Considerable effort has been focused on methods to synthesize these nanocarriers in the past decades. However, most methods fail to controllably generate lipid vesicles at specific sizes and with low polydispersity, especially via scalable approaches suitable for clinical product manufacturing. Here, we report a surfactant-assisted liposome assembly method enabling the precise production of monodisperse liposomes with diameters ranging from 50 nm to 1 μm. To overcome scalability limitations, we used tangential flow filtration, a scalable size-based separation technique, to readily concentrate and purify the liposomal samples from more than 99.9% of detergent. Further, we propose two modes of liposome self-assembly following detergent dilution to explain the wide range of liposome size control, one in which phase separation into lipid-rich and detergent-rich phases drives the formation of large bilayer liposomes and a second where the rate of detergent monomer partitioning into solution controls bilayer leaflet imbalances that promote fusion into larger vesicles. We demonstrate the utility of controlled size assembly of liposomes by evaluating nanoparticle uptake in macrophages, where we observe a clear linear relationship between vesicle size and total nanoparticle uptake.

## Introduction

Lipid-based nanoparticles, such as liposomes, are effective vehicles for the delivery of therapeutic agents. Since their first Food and Drug Administration approval in 1995 for the drug Doxil,^1^ the development of these nanocarriers has greatly expanded owing to their ability to efficiently package therapeutics and improve accumulation in target tissues.^2,3^ While there are many strategies to generate lipid vesicles, most methods involve kinetically trapped species, which often limits control over their size and polydispersity.^4−6^ Moreover, the requirement of rapid mixing in many of these systems presents a significant challenge to scale-up manufacturing.^4^

One of the earlier reported methods to generate liposomes is based on solubilizing lipid components into detergent micelles followed by either dialysis or gel filtration chromatography to remove detergent.^6,7^ While this method is still commonly used for proteoliposome synthesis,^8^ for laboratory studies this approach has been largely displaced by solvent dispersion or thin film hydration methods that do not require tedious and time-consuming purification steps.^6,9^ However, advancements in size-exclusion methods, such as tangential flow filtration (TFF), have made size-based separations a faster and scalable process.^10^ Indeed, we previously showed that dilution of mixed detergent/lipid micelles followed by TFF enabled controlled self-assembly of immune-stimulating complexes that can be readily produced at clinical lot scales.^11^

We hypothesized that an improved method to manipulate lipid self-assembly from detergent micelles could enable liposome generation with more precise particle size control and lower polydispersity relative to commonly used approaches. In this study, we revisited the detergent removal method for liposome synthesis using TFF for rapid concentration of samples and removal of detergent and determined key parameters controlling liposome self-assembly. Vesicle formation was induced by diluting a lipid/detergent mixture (mixed micelles) below the critical micelle concentration (CMC) of the detergent. We discovered that the concentration of detergent during self-assembly allowed for precise control of liposome size from ∼1 μm down to 50 nm with low polydispersity [polydispersity indices (PDIs) below 0.1]. To understand this process, we developed a model for the growth of liposomes based on phase separation of lipid-rich and detergent-rich phases in solution during detergent removal that enabled large liposome assembly. Finally, we demonstrate the utility of controlled liposome assembly by validating that liposomes produced by this process are differentially recognized by macrophages as a function of the particle size.

## Experimental Section

### Materials

1,2-Distearoyl-*sn*-glycero-3-phosphocholine (DSPC), cholesterol (chol), 1-palmitoyl-2-oleoyl-*sn*-glycero-3-phospho-(1′-rac-glycerol) (sodium salt) (POPG), 1,2-dioleoyl-*sn*-glycero-3-phosphoethanolamine-*N*-(glutaryl) (sodium salt) (DOPE-glutaryl), 1,2-dioleoyl-*sn*-glycero-3-phosphoethanolamine-*N*-dibenzocyclooctyl (DOPE-DBCO), and 1,2-distearoyl-*sn*-glycero-3-phospho-(1′-rac-glycerol) (DSPG) were purchased from Avanti polar lipids. *N*-Decanoyl-*N*-methylglucamine (MEGA-10) and *n*-octyl-β-d-glucoside (octylglucoside) were purchased from Sigma-Aldrich. For fluorescence measurements, borondipyrromethene (BDP) tetramethylrhodamine (TMR) azide (Lumiprobe) and BDP 630/650 azide (Lumiprobe) were reacted with DOPE-DBCO in chloroform to generate DOPE-TMR and DOPE-630/650. Successful conjugation was validated via thin-layer chromatography which indicated <1% free dye.

### Generation of Detergent/Lipid Mixtures

Lipid stock solutions were made in chloroform and then measured into glass vials and left to dry on a desiccator overnight. For solubilization in MEGA-10 micelles, a 10% MEGA-10 solution was dissolved in deionized water. The 10% MEGA-10 solution was then added to the dried lipids and left in a water bath sonicator at 50–60 °C until all lipids were solubilized. The lipid/detergent mixture was allowed to equilibrate at 25 °C prior to dilution. The same process was followed for experiments using octylglucoside instead of MEGA-10.

### Synthesis of Nanoparticles Via Dilution

Lipid/detergent micelles were diluted by adding buffer to the micelles to reach the target detergent concentration. For small-scale samples (<2 mL), the mixed micelle mixture was added to a container, and then buffer was added and mixed with a 1 mL pipet. No major difference in particle size was observed when mixed micelles were directly added to the buffer instead. For larger scales (>2 mL), the mixed micelles were added to the bottom of a container that could house the full dilution volume. Then, the buffer was added to the sides of the vessel with a serological pipet gun while the sample was swirled. Upon full dilution, the sample was further mixed with the serological pipet gun. The samples were then allowed to equilibrate at 25 °C.

### Purification Via TFF

Generally, 5 mg of the lipid/detergent mixtures was diluted to the target MEGA-10 concentration and left to self-assemble overnight. Then, samples were diluted to 0.02% MEGA-10 to ensure minimal effects of the detergent on the assembled lipid structures. Samples were then placed on a KrosFlo KR2i TFF system (Repligen) using a 100 kDa mPES membrane with a surface area of 115 cm^2^. Samples underwent 10 diafiltration volumes against the buffer used for their assembly. Particle yield was either accessed based on recovered nanoparticle fluorescence or total lipid content measured via the Steward assay.^12^ For cryogenic transmission electron microscopy (cryo-TEM) analysis, samples underwent 5 diafiltration volumes against deionized water to remove salts.

### Analysis of Lipid Transfer Via Fluorescence Resonance Energy Transfer (FRET)

Lipid/detergent micelles of the desired composition were generated by replacing 1 mol % of DSPC with 1 mol % of either DOPE-TMR (donor micelles) or DOPE-630/650 (acceptor micelles). For FRET micelles, donor micelles and acceptor micelles were mixed in a 1:1 ratio. FRET micelles, donor micelles, and acceptor micelles were diluted to the target MEGA-10 concentration. After dilution of donor and acceptor micelles, the diluted samples of equal final MEGA-10 concentration were mixed at 0, 1, and 24 h after dilution. FRET efficiency was then measured at 0, 1, 24, and 48 h after mixing of donor and acceptor micelles. Fluorescence values were measured in a 384-well plate in a Tecan Infinite 200. FRET efficiency was calculated based on the equation for the corrected FRET efficiency (FRET_N_) described previously.^13^ Briefly, acceptor-only and donor-only controls were evaluated for their emission in the donor (530 nm excitation/570 nm emission), acceptor (610 nm excitation/650 nm emission), and FRET (530 nm excitation/650 nm emission) channels. For FRET efficiency measurements, the acceptor fluorescence channel was used to correct for the donor channel fluorescence. Then, both acceptor channel fluorescence and corrected donor channel fluorescence were used to correct the FRET channel fluorescence for any contribution of donor or acceptor emission in the channel, so that only energy transfer was measured. FRET efficiency was then calculated based on the ratio of corrected FRET channel measurement relative to the corrected donor channel fluorescence.

### Characterization of Particle Preparations

Dynamic light scattering (DLS) and zeta potential measurements were made on a Zetasizer Nano ZSP instrument (Malvern). Nanoparticle micrographs were acquired using cryo-TEM on a JEOL 2100 FEG microscope (200 kV). For cryo-TEM, particles were buffer exchanged into deionized water via either dialysis or TFF unless otherwise indicated. For grid preparation, 3 μL of the sample was dropped on a lacey copper grid coated with a continuous carbon film and blotted to remove excess sample without damaging the carbon layer by a Gatan Cryo Plunge III. The grid was then mounted on a Gatan 626 single-tilt cryo-holder equipped in the TEM column. The specimen and holder tip were frozen and cooled by liquid nitrogen, and the temperature was maintained during transfer into the microscope and subsequent imaging. Images were collected with a magnification range of 10,000–60,000×. Cryo-TEM micrographs were analyzed on ImageJ to measure particle diameter. PDI from diameter distribution was determined based on the PDI equation used for DLS: , where is σ the standard deviation of the particle diameter and μ is the average diameter.

### Cell Culture

RAW 264.7 macrophages (ATCC) were cultured in DMEM. Cell media were supplemented with 10% FBS and penicillin/streptomycin with cells incubated in a 5% CO_2_ humidified atmosphere at 37 °C. All cell lines were murine pathogen tested and confirmed mycoplasma negative by a Lonza MycoAlert Mycoplasma Detection Kit.

### In Vitro Cellular Association

Liposomes were generated with 0.2 mol % of DOPE-630/650, which is composed of a dye relatively insensitive to the polarity and pH of the environment.^14^ The day before dosing, RAW 264.7 cells were plated on a tissue-culture 96-well plate at a density of 25,000 cells per well. The next day, wells were dosed with liposomes at 0.01 mg/mL and left for the target incubation time (4 or 24 h). To determine the percentage of liposomes associated with macrophages, a sample of the supernatant was removed from the well and diluted 5× with dimethyl sulfoxide (DMSO). Cells were then washed three times with phosphate-buffered saline (PBS) and scraped and dissolved with DMSO to homogenize and extract all fluorescent lipids from the cell. Fluorescence of the liposomes remaining in the supernatant and fluorescence of liposomes associated with macrophages were then measured using a fluorescence plate reader (TECAN Infinite 200).

## Results and Discussion

### Concentration of Detergent Enables Precise Control of Varied Liposome Size with Low Polydispersity

**Figure 1:**
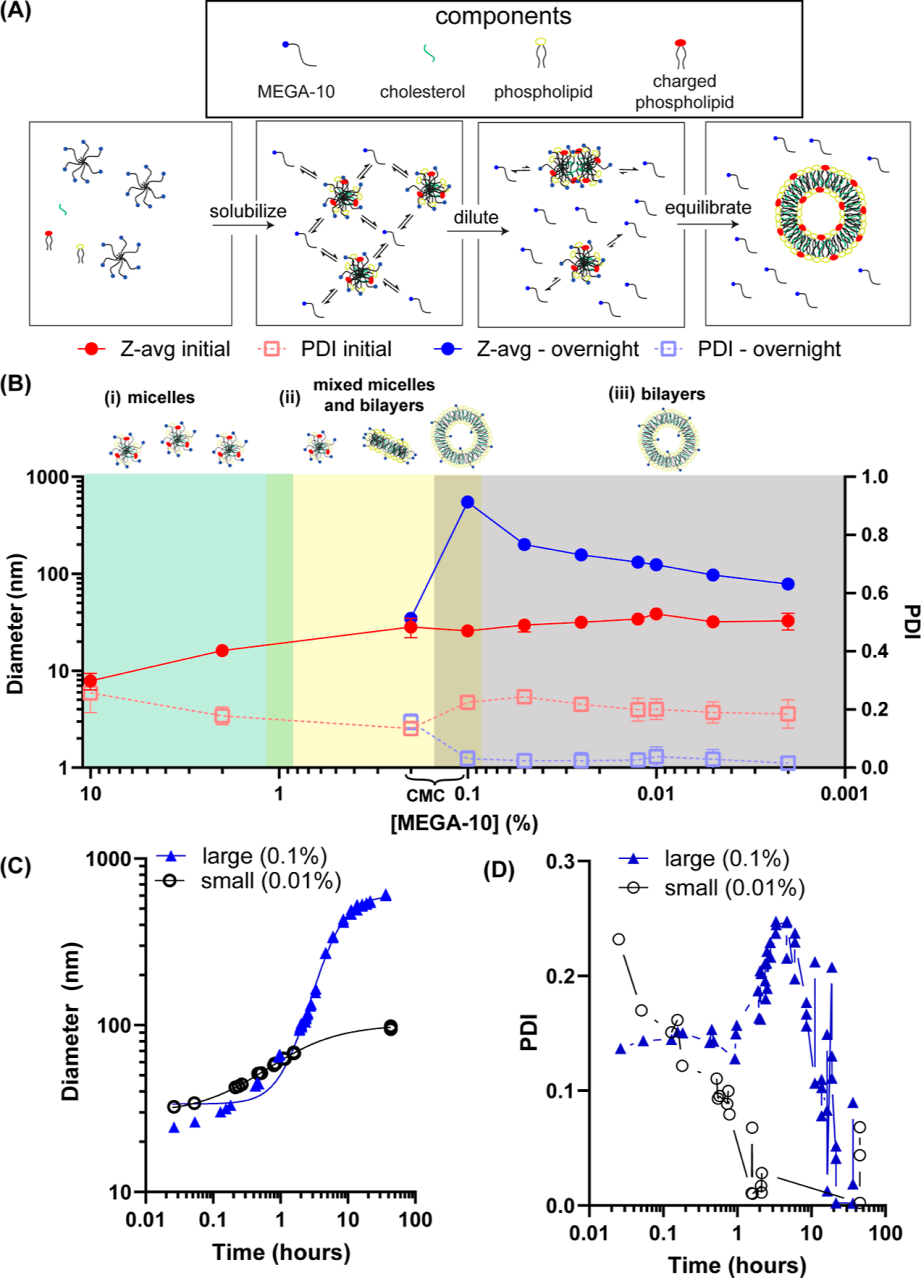
Detergent concentration during nanoparticle formation dictates the equilibrium size of nanoparticles. (A) Schematic of liposome assembly from mixed micelle dilution. (B) Particle size (Z-avg) determined by DLS of samples diluted with PBS to various final concentrations of MEGA-10 starting from an initial mixture of 10 mg/mL 6:3:1 molar mixture of DSPC/chol/POPG in 10% MEGA-10. (C) Particle size evolution over time determined by DLS for mixed micelles diluted to 0.1 and 0.01% MEGA-10. Solid lines represent fits of the data to the Hill Equation. (D) PDI kinetics for mixed micelles diluted to 0.1 and 0.01% MEGA-10.

Liposome assembly from mixed micelles derived from surfactants and lipids occurs by depleting detergent molecules from micellar structures, leading to lipid aggregate coalescence into bilayer vesicles.^15,16^ Prior studies on liposome formation from nonionic detergent micelles have focused on the rate of detergent removal from the sample to control the final liposome size.^7,17^ We hypothesized that the final detergent concentration could control self-assembly due to both the kinetics and the equilibrium partitioning of the detergent into the aqueous phase. To test this idea, we evaluated liposome assemblies formed following rapid dilution of lipid/detergent micelles followed by overnight equilibration at different final total detergent concentrations (Figure 1A). As a model system, we used lipid mixtures with a 6:3:1 molar ratio of DSPC, chol, and POPG as we and others have used similar formulations for therapeutic liposomes (see component structures in Figure S1).^18−22^ The detergent, MEGA-10, at an initial concentration of 10 wt % was chosen due to its high CMC (∼0.2–0.1%^23^), which facilitates surfactant removal; and samples were diluted with pH 7.4 PBS, a physiological buffer. After rapid dilution of MEGA-10/lipid solutions to varying final detergent concentrations, the sample size and PDI were measured via DLS before and after overnight incubation to ensure final self-assembly.

Immediately following dilution, no major changes in particle size were observed even at detergent concentrations far below the CMC (Figure 1B, Z-avg initial). However, after 18 h of incubation at dilutions below the detergent CMC, particles were detected by DLS with mean sizes ranging from 50 to 500 nm (Figure 1B, Z-avg overnight). Particle size was determined by the final concentration of MEGA-10, even though no detergent molecules were removed, and all samples were diluted at the same rate. Unexpectedly, we were able to achieve more than 1 order of magnitude variation in final particle size with very low polydispersity. We confirmed that these resulting particles were spherical unilamellar liposomes via cryo-TEM (Figure S2). Previous work on mixed micelles has suggested the presence of three phases during the transition between micelles to vesicles that help explain the observed size changes.^24^ At high concentrations of detergent, a small spherical micelle-predominant region exists [region (i)] that transitions into a mixed micelle region [region (ii)], followed by a final bilayer-predominant (vesicle) region at detergent concentrations below the CMC [region (iii)]. In region ii, mixed micelle structures may include disc-like bicelles or cylindrical worm-like micelles that coexist with vesicles.^15^

Given the unusually low polydispersity when large (>200 nm) particles were formed near the detergent CMC, we first explored the limits of final vesicle size.^25^ By slightly increasing the MEGA-10 concentration from 0.1% MEGA-10, it was possible to achieve >1 μm particles with PDIs below 0.1, indicative of a monodisperse population (Figure S3A).^19^ To validate that this observed size control was not restricted to MEGA-10, we tested the same protocol using a different nonionic surfactant, *n*-octyl-β-d-glucoside (octylglucoside, CMC ∼ 0.6–0.7%). Like MEGA-10 mixed micelle dilution, we observed the three distinct regions of lipid assembly and the formation of large monodisperse particles below the CMC (Figure S3B). Moreover, to ensure that the particle size was not dependent on residual detergent in the bilayer, we further diluted equilibrated samples and found no change in size or polydispersity, suggesting that stable lipid assemblies had been formed (Figure S3C).

We next characterized the kinetics of lipid vesicle assembly at 0.1% MEGA-10 and compared it to that of smaller vesicles formed at 0.01% MEGA-10. While self-assembly at 0.01% MEGA-10 was rapid, reaching near final sizes within ∼3 h, self-assembly at 0.1% MEGA-10 required >10 h to approach equilibrium (Figure 1C). The observed kinetic curves were well fit by the Hill equation, which indicated cooperative assembly (*n* > 1^26^) at 0.1% MEGA-10 (*n* ∼ 2), but not at 0.01% MEGA-10 (*n* = 0.9).^22^ Further, while the PDI of particles formed at 0.01% MEGA-10 decreased monotonically with time, at 0.1% MEGA-10, a transient transition into a polydisperse sample was observed (Figure 1D), and the PDI did not begin decreasing until ∼10 h of incubation for self-assembly had passed. We hypothesize this decrease in PDI during overnight incubation represents a gradual transition of small heterogeneous mixed micelles into uniform lipid vesicles as these lipid assemblies slowly equilibrate.

### Purification of Assembled Nanoparticles Via TFF Removes Detergent without Affecting Liposome Structure

**Figure 2:**
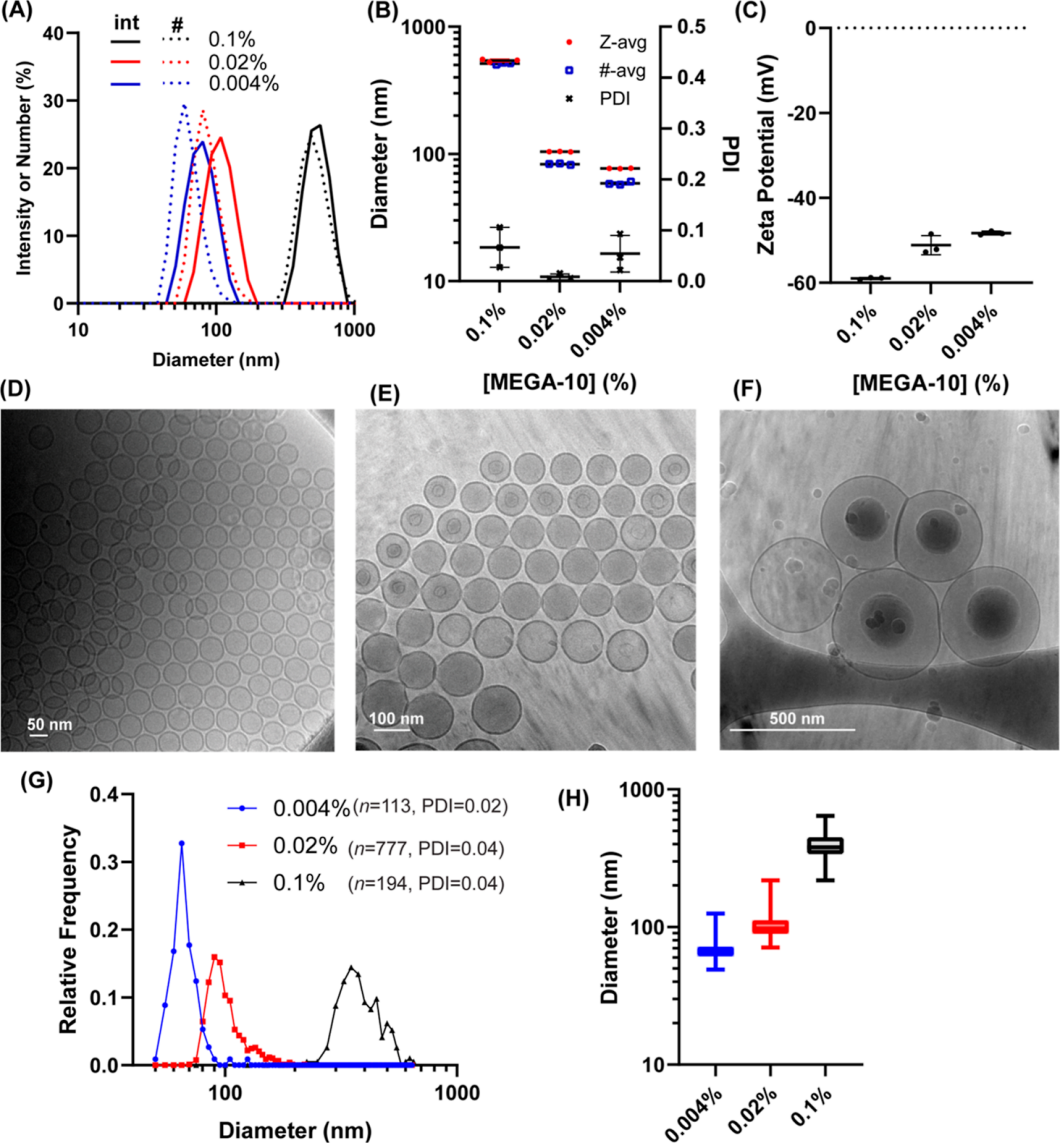
Purified lipid nanoparticles maintain size and monodispersity. (A) DLS intensity and number distribution for liposomes generated by diluting a lipid mix (10 mg/mL 6:3:1 molar mixture of DSPC/chol/POPG in 10% MEGA-10) to 0.1, 0.02, and 0.004% of detergent. (B) Size and PDI of samples from (A). (C) Zeta potential of samples from (A)—the variation in measured zeta potentials can be explained by the approximation of the Henry’s function as a constant in our instrument given that for a given zeta potential, increased particle size increases electrophoretic mobility.^27^ Representative cryo-TEM images of samples from samples generated at (D) 0.004%, (E) 0.02%, (F) and 0.1%—internal shading on large liposomes are due to particle protrusion from ice.^28^ (G) Histograms of particles measured on cryo-TEM micrographs from samples in (A)—parentheses indicates the total number of particles quantified and PDI based on the measured particle sizes from cryo-TEM. (H) Box and whiskers plot of size distribution from (G).

Having observed an unexpected wide range of control over monodisperse liposome assembly using detergent dilution, we next sought to assess removal of the surfactant using TFF as residual detergent could be a potential source of toxicity and interfere with liposome behavior. In addition, TFF provides a convenient means by which to concentrate the particles. We tested purification of samples equilibrated at 0.1, 0.02, or 0.004% MEGA-10 to form vesicles of three distinct sizes (Figure 2A,B). Detergent was removed by concentrating the samples to 0.25 mg/mL lipids and then purifying via 10 diafiltration volumes through a 100 kDa MWCO TFF membrane. Analysis of the purified vesicles via reverse-phase high-pressure liquid-chromatography coupled with evaporative light scattering detector showed no detectable levels of MEGA-10 (<1% mass composition out of the total lipid components), indicating removal of greater than 99.9% of MEGA-10 from the sample (Figure S4A,B). We also found no appreciable difference in the final particle size of small-scale (∼0.05 mg) or larger batches (5 mg) prepared by this process, and particles maintained their size and monodispersity with yields of 70–80% after purification (data not shown). Zeta potential measurements indicated the expected negative surface charge on these particles due to the presence of POPG (Figure 2C). Analysis via cryo-TEM revealed that the vesicles were primarily unilamellar liposomes with a narrow polydispersity (Figure 2D–H). Compared to conventional thin-film hydration followed by extrusion through a 50 nm pore-sized membrane (Figure S5A,D), even the large liposomes of ∼500 nm mean diameter had lower polydispersity when preparted via detergent dilution and TFF purification (Figure S5E,F).

### Electrostatic Interactions and Lipid Membrane Rigidity Enable Controlled Growth of Liposomes

**Figure 3:**
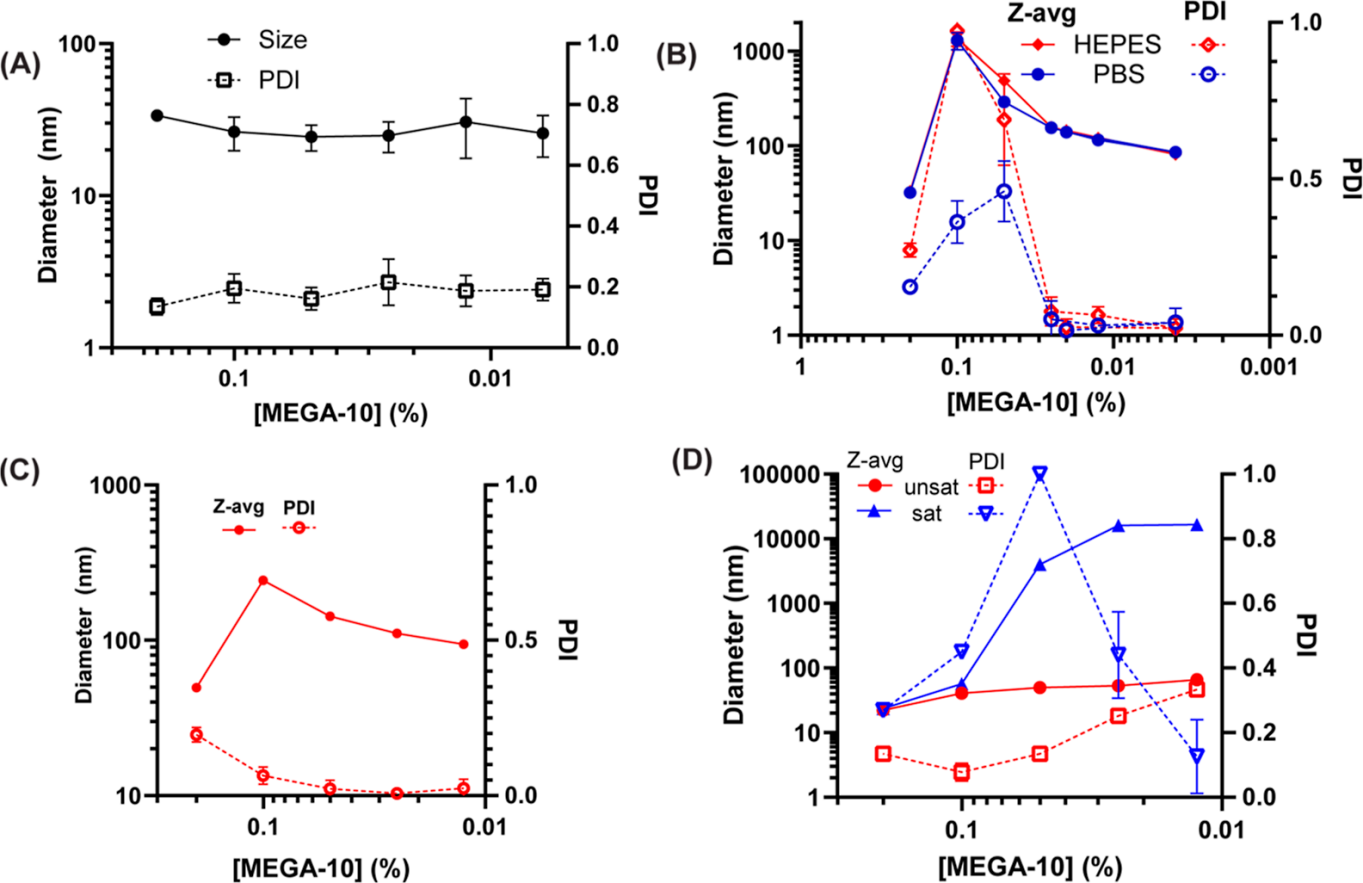
Solution ionic strength and lipid composition regulate the self-assembly from lipid/detergent micelles into liposomes. (A) Particle size (Z-avg) and PDI after lipid/detergent micelle dilution (10 mg/mL 6:3:1 molar mixture of DSPC/chol/POPG in 10% MEGA-10) with 10 mM HEPES and overnight incubation. (B) Z-avg and PDI of neutral lipid/detergent micelles (10 mg/mL 7:3 molar mixture of DSPC/chol in 10% MEGA-10) diluted with either PBS or 10 mM HEPES. (C) Z-avg and PDI of anionic lipid/detergent micelles charged with DSPG either containing or lacking chol (10 mg/mL 6:3:1 or 9:1 molar mixture of DSPC/chol/DPSG or DSPC/DSPG in 10% MEGA-10) diluted with PBS. (D) Z-avg and PDI of chol-free unsat mixed micelles (9:1 DSPC/POPG) and sat mixed micelles (9:1 DSPC/DSPG) diluted with PBS to various final MEGA-10 concentrations and allowed to incubate overnight at room temperature.

To explore the system characteristics that enabled a wide range of liposome size control, we first assessed whether the ionic strength of the dilution buffer affected equilibrated particle size by carrying out rapid dilution to varying concentrations of detergent using a lower ionic strength buffer (10 mM HEPES). Under these low ionic strength conditions, there was no appreciable change in the size of mixed micelles regardless of final detergent concentration over 18 h (Figure 3A), indicating that the high electrostatic repulsion between micelles limited the growth of larger species. To test whether electrostatic interactions played a role in achieving monodisperse vesicle assembly, we evaluated liposome assembly from mixed micelles lacking the charged POPG lipid (i.e., neutral DSPC/chol liposomes) in PBS or HEPES. With this formulation, it was possible to generate small vesicles with low polydispersity using both buffers, and the transition from small mixed micelles to larger particles occurred at similar MEGA-10 concentrations (0.2–0.1 wt %, Figure 3B). However, the size control was limited as vesicles >200 nm in diameter formed at lower MEGA-10 dilutions had high polydispersity, indicating that a balance of charge repulsion and particle coalescence was important for the controlled formation of large monodisperse vesicles (Figure 3B).

As lipid unsaturation and the presence of chol are known to promote liposome fusion as well as alter lipid bilayer rigidity,^29,30^ we next evaluated the effect of lipid composition by removing chol or replacing POPG with DSPG, a saturated (sat) anionic lipid. Dilution of DSPG/DSPC/chol mixed micelles with either PBS or HEPES-only buffer behaved like POPG/DSPC/chol mixed micelles (Figure 3C) but with lower overall liposome sizes. On the other hand, removal of chol from the unsaturated (unsat) formulation (DSPC/POPG mixture only) fully prevented the formation of large vesicles at 0.1% MEGA-10 and yielded polydisperse samples at low MEGA-10 concentrations (Figure 3D). Dilution of sat lipid compositions devoid of chol yielded only large polydisperse species (Figure 3D). Thus, control over liposome size required the presence of chol and was facilitated by lipid unsaturation, consistent with properties that promote bilayer fusion.

### Self-Assembly of Large Liposomes Occurs through Phase Separation into Lipid-Rich and Detergent-Rich Phases

**Figure 4:**
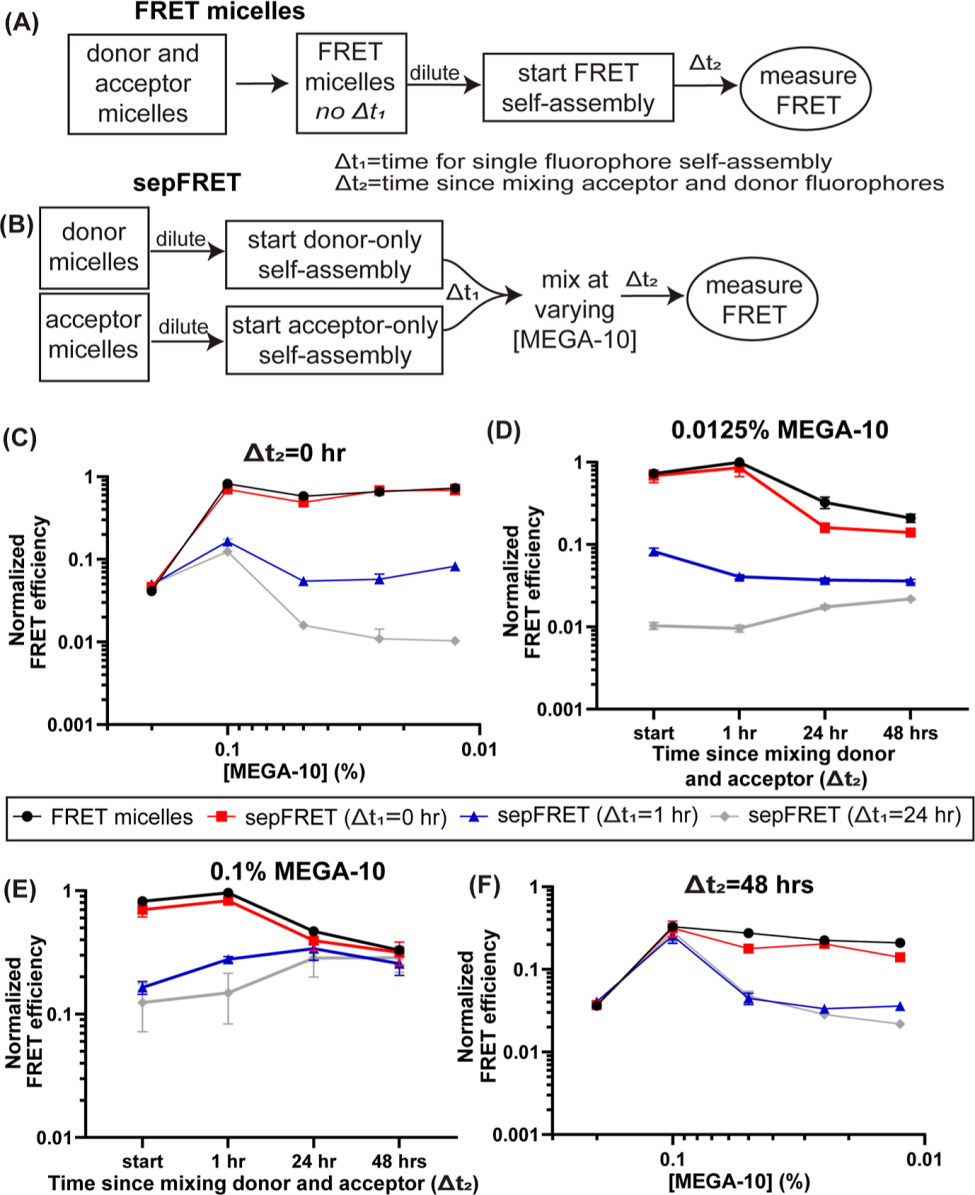
FRET of diluted mixed micelles reveals intermediates with high rates of lipid exchange. (A) Schematic for the dilution of FRET micelles. (B) Schematic for separately diluting donor-only or acceptor-only micelles and then mixing the diluted samples (sepFRET). (C) Normalized FRET efficiency of FRET micelles and sepFRET micelles right after mixing donor and acceptor (0 h). (D) Time course of normalized FRET efficiency since mixing donor and acceptor micelles at 0.0125% MEGA-10 for FRET micelles and sepFRET micelles. (E) Time course of normalized FRET efficiency since mixing donor and acceptor micelles at 0.1% MEGA-10 for FRET micelles and sepFRET micelles. (F) Normalized FRET efficiency of FRET micelles and sepFRET micelles 2 days after mixing donor and acceptor (48 h).

Given the possibility of micelle and bilayer coexistence near the detergent’s CMC of mixed micelles,^24,25^ which should result in enhanced lipid transfer between both bilayers and micelles,^31,32^ we turned to the use of FRET to evaluate if lipid exchange was occurring during vesicle self-assembly at various detergent concentrations. We first evaluated FRET during self-assembly from mixed micelles (6:3:1 molar ratio of DSPC/chol/POPG) containing both donor and acceptor fluorescently tagged lipids (FRET micelles, Figure 4A). To assess if lipid mixing could occur at various stages of vesicle self-assembly, we separately diluted mixed micelles containing either donor-only or acceptor-only fluorescently tagged lipids (sepFRET micelles). After a given time for self-assembly, samples at the same concentration of MEGA-10 were mixed to evaluate changes in the FRET efficiency (Figure 4B).

When donor and acceptor micelles were mixed at 10% MEGA-10, a low energy transfer was measured (∼0.05 normalized FRET efficiency). Yet when FRET micelles were diluted to 0.2% MEGA-10 or lower, a marked increase in FRET efficiency was observed (Figures 4C and S6). Further, sepFRET micelles behaved similarly to FRET micelles when donors and acceptors were mixed right after dilution (sepFRET-0 h), indicating that the initial intermediates generated upon dilution undergo rapid lipid mixing during self-assembly. However, after 1 h or more of independent self-assembly of donor and acceptor samples (sepFRET >1 h), no major increase in FRET efficiency could be seen below 0.1% MEGA-10, indicating that the later intermediates in region (iii) did not undergo rapid lipid mixing events. Indeed, sepFRET samples allowed to assemble for one or more hours maintained low FRET efficiency for 48 h, which is consistent with the expected low lipid exchange rate of assembled liposomes (Figure 4D). However, at 0.1% MEGA-10, all sepFRET groups converged to similar FRET efficiency levels as that of FRET micelles (Figure 4E), which was not observed for lower concentrations of MEGA-10 (Figure 4F). The high rate of lipid exchange at 0.1% MEGA-10 after 24 h of sepFRET assembly suggested that the large species formed at 0.1% MEGA-10 were in a state of dynamic equilibrium likely due to the coexistence of mixed micelles and bilayers.

**Figure 5:**
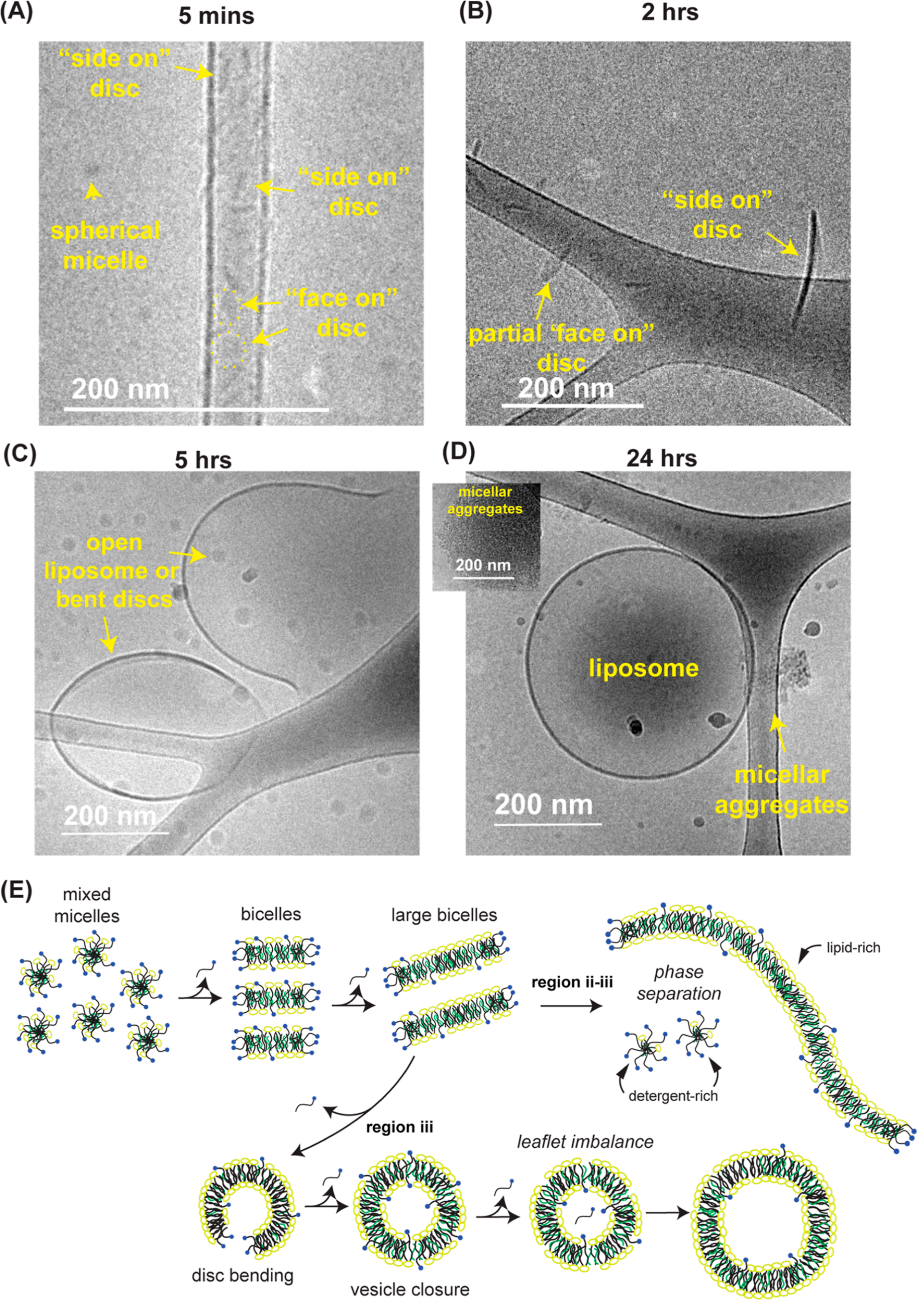
Cryo-TEM micrographs of particles incubated in 0.1% MEGA-10 reveal the formation of large disc assemblies and the coexistence of micelles with fully assembled bilayer vesicles. (A–D) Cryo-TEM micrograph of 10 mg/mL 6:3:1 DSPC/chol/POPG sample in 10% MEGA-10 rapidly diluted to 0.1% MEGA-10 with 10 mM HEPES and 150 mM NaCl and frozen 5 min, 2, 5, and 24 h after dilution, respectively. (E) Schematic for the two proposed main driving mechanisms of particle coalescence and growth upon mixed micelle dilution.

We next used cryo-TEM to visualize intermediates that allowed for the assembly of large liposomes. We diluted mixed micelles with 10 mM HEPES and 150 mM NaCl (HEPES was used instead of PBS as phosphates can interfere with cryo-TEM imaging) to 0.1% MEGA-10 and processed samples for cryo-TEM analysis after 5 min, 2, 5, or 24 h of incubation at 25 °C. Upon dilution, the sample started as small (∼15 nm) spherical micelles and disc-like or worm-like structures (Figures 5A and S7A) that accumulated at the carbon region of the TEM grid likely due to their small size and low concentration.^33^ These small micellar structures coalesced to larger (>100 nm) bilayer discs and liposomes within 1–2 h (Figures 5B and S7B). At 5 h, smaller discs were no longer seen, and the sample was composed of primarily large (>300 nm) discs and liposomes (Figures 5C and S7C). Finally, after 24 h, only large liposomal species could be seen as well as micellar aggregates^34^ (Figures 5D and S7D). The presence of these micellar aggregates in cryo-TEM micrographs confirmed the expectation of a mixed micelle coexistence with bilayers.

Based on these results, we proposed two distinct mechanisms for particle coalescence and growth (Figure 5E). When diluted to the interface between region ii and region iii, where bilayers and mixed micelles may coexist (∼0.1% MEGA-10, see Figure 1B), bicelles (surfactant-stabilized bilayer nanodiscs) initially form. As further detergent partitions into the aqueous phase, bicelle fusion is driven as less detergent is available to stabilize the outer edges of discoidal assemblies. However, when the detergent monomers begin to equilibrate with the aqueous phase, there is still sufficient detergent incorporated into the bilayers to prevent complete stable vesicle closure. Instead, the detergent partitions into lipid-rich and detergent-rich phases driven by the curvature preference of the surfactant molecules. As large bilayer discs start to form, less detergent is freed from the discoidal rims upon further fusion and charge repulsion between particles increases, enabling the system to reach a terminal size.

If vesicles are formed prior to detergent equilibration with the aqueous phase, detergents released into the internal aqueous volume of the liposome cannot freely diffuse to the external volume, whereas outer leaflet detergent molecules are continuously depleted, leading to an imbalance on the required number of molecules between the inner and outer leaflet (Figure 5E).^15,35^ These imbalances are known to promote fusion or vesicle rupture, and its effect becomes more pronounced as the size of the vesicle grows.^35^ This leaflet imbalance-driven particle coalescence is likely the primary cause for liposome fusion when diluted to lower detergent concentrations of region iii due to rapid detergent depletion from bilayers (i.e., lack of detergent-stabilized bicelles) and lack of mixed micelle coexistence.^15^ Under this scenario, the final liposome size is dictated by the number of fusion events required to normalize the leaflet imbalances such that higher dilutions increase the rate of detergent partitioning into the aqueous phase prior to vesicle closure and, subsequently, smaller liposome size.

### Purified Liposomes Maintain Expected Biophysical Interactions with Macrophages

Different-sized nanoparticles have different biophysical interactions with cells. Thus, we next wanted to validate that the method for liposome assembly presented here can be used to probe such interactions. While conflicting results have been presented,^36^ most studies have indicated that macrophages preferentially uptake larger liposomes in vitro.^37−40^ Unlike most normal cells, macrophages are known as professional phagocytes, which enable them to efficiently uptake particles with diameters above 200 nm.^41,42^ However, prior work has failed to present a clear relationship between liposome size and uptake, potentially due to the use of thin film hydration followed by extrusion, where it is difficult to control the liposome size and lamellarity. Given that the method presented here enabled the synthesis of large and monodisperse unilamellar liposomes, we decided to evaluate the effect of the liposome size on macrophage uptake.

**Figure 6:**
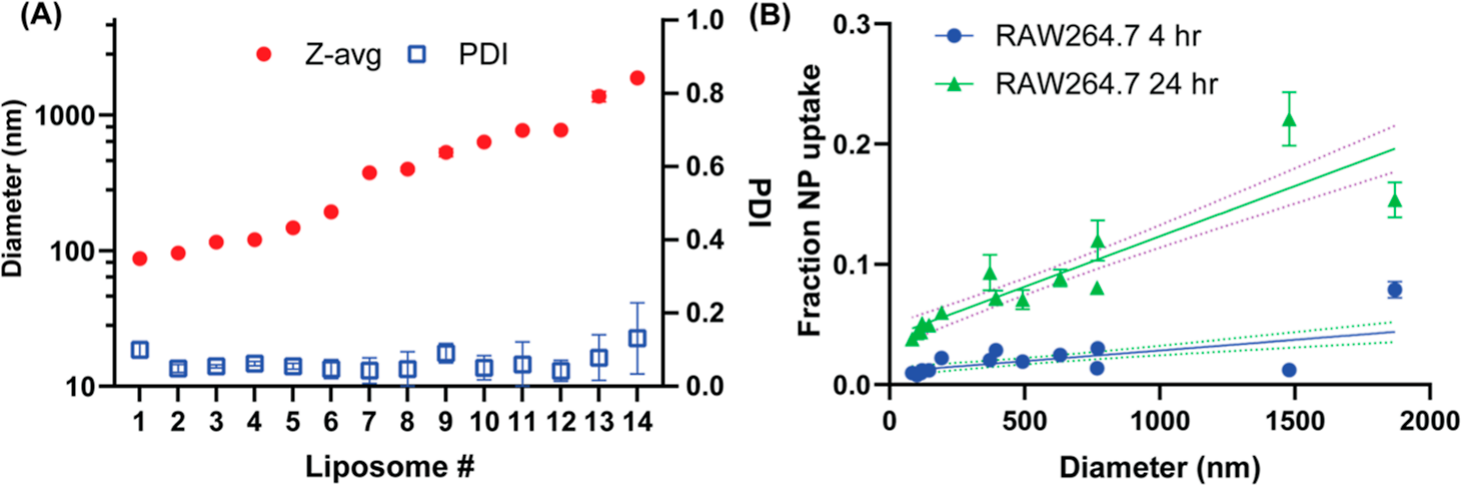
Controlled assembly of liposomes reveals a linear size-dependence effect on NP uptake in macrophages in vitro. (A) Intensity weighted size (Z-avg) and PDI of liposomes generated for this experiment. (B) NP uptake on RAW264.7 macrophage cells after 4 or 24 h of incubation with NPs. Dashed lines indicate 95% confidence interval of linear fit.

To investigate this trend, we first generated a library of varied-sized liposomes, comprised of 14 samples with hydrodynamic sizes (Z-avg) spanning from ∼100 nm diameter to ∼1 μm, all with low polydispersity—this size series demonstrates the high degree of control that this process provides over vesicle generation (Figure 6A). We then dosed RAW 264.7 macrophages with equal mass concentrations of each particle (i.e., equal amounts of total fluorescence lipids). As equal masses of lipids were dosed, increasing the particle size effectively reduced the number of liposomes per well but maintained the total particle surface area as liposomes are 2D assemblies. After 4 or 24 h of incubation, the remaining liposome signal in the supernatant and liposome signal associated with macrophages were measured to determine a percentage of vesicle uptake (Figure S8). As expected, macrophages were found to have an increased uptake of large liposomes (Figure 6A). Importantly, however, through our assembly method, we could see that there was a clear linear relationship between liposome size and liposome uptake at either 4 or 24 h of incubation (Figure 6B). Thus, the generation of monodisperse liposomes allows biological interactions with cells to be more clearly assessed.

## Conclusions

Liposomes are important delivery vehicles for both the current and the next generation of therapeutics. Here, we show that, through the rational disassembly of mixed micelles, we can precisely control the size of charged liposomes.^15^ Although previous work using glycocholate and egg phosphatidylcholine similarly found that dilution of micellar mixtures could control liposome size, these studies only achieved size ranges of only ∼50–100 nm.^25,43^

Through a combined assessment of the assembly kinetics, lipid composition effects, and lipid exchange rates at each detergent concentration, we were able to propose qualitative models to describe the size control, which has been lacking in the literature.^15^ The models combine and build upon prior work, which has described phase separation at the transition between mixed micelles and bilayer,^15,24^ the potential for detergent entrapment in the inner core of vesicles upon disc closure,^24^ and how leaflet imbalances induce bilayer fusion.^35^

Furthermore, while our results contradict the idea that the rate of detergent removal either via controlled dilution or controlled dialysis governs final liposome size,^7,17,44,45^ the rate of detergent removal likely alters the residence time of the lipidic mixture at each concentration of detergent, leading to the control in liposome size observed previously. Future studies are also needed to validate the models presented here as we are unable to make conclusions on the system reaching full chemical equilibrium.

Taken together, the findings presented here demonstrate a promising new method of detergent-aided assembly of monodisperse liposomes with controlled, scalable techniques. The benefits of generating particles across a wide range of sizes with little variance open the doors to accurate investigations of the effect of liposome size in biological systems. Further, the techniques and insights presented may facilitate the generation of new and more controlled lipid-based assemblies.

## Data Availability

Raw data are available from the corresponding author upon request.

## Supporting Information Available

Additional information on chemical structure of components used in the experiments, cryo-TEM of liposomes formed upon detergent dilution, comparison of MEGA-10 or octylglucoside mixed micelle dilutions and stability of assembled particles upon further dilution, characterization of residual detergent after purification, full data set on FRET signal upon mixed micelle dilution, extra cryo-TEM images during lipid self-assembly at 0.1% MEGA-10, and diagram of experimental protocol for in vitro NP uptake assay (PDF)
