# Supplementary material for: Surfactant-Mediated Assembly of Precision-Size Liposomes
Source: Chem Mater. 2024 Jul 25;36(15):7263–73. doi: 10.1021/acs.chemmater.4c01127 (PMC11325547; doi:10.1021/acs.chemmater.4c01127)
Supplement: Supplementary file 1 — cm4c01127_si_001.pdf [file cm4c01127_si_001.pdf]

Supporting Information for

Surfactant-mediated assembly of precision-size liposomes

Ivan S. Pires[a,b], *Jack R. Suggs*[a], *Isabella S. Carlo*[a], *DongSoo Yun*[a], *Paula T. Hammond*[a,b,*], *Darrell J. Irvine*[a,c,d,e,f,*]

[a]Koch Institute for Integrative Cancer Research, Massachusetts Institute of Technology, 500 Main Street, Cambridge, Massachusetts 02139, United States

[b]Department of Chemical Engineering, Massachusetts Institute of Technology, 21 Ames Street, Cambridge, Massachusetts 02139, United States

[c]Department of Biological Engineering, Massachusetts Institute of Technology, 25 Ames Street, Cambridge, Massachusetts 02139, United States

[d]Department of Materials Science and Engineering, Massachusetts Institute of Technology, Cambridge, MA 02139, USA

[e]Ragon Institute of Massachusetts General Hospital, Massachusetts Institute of Technology and Harvard University, Cambridge, MA 02139, USA

[f]Howard Hughes Medical Institute, Chevy Chase, MD 20815, USA

*Email: hammond@mit.edu (Paula T. Hammond)

*Email: djirvine@mit.edu (Darrell J. Irvine)

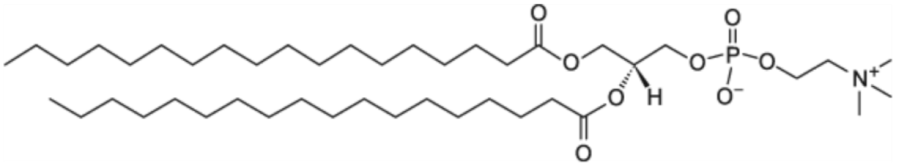

1,2-distearoyl-sn-glycero-3-phosphocholine (**DPSC**)

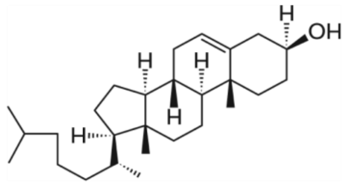

cholesterol

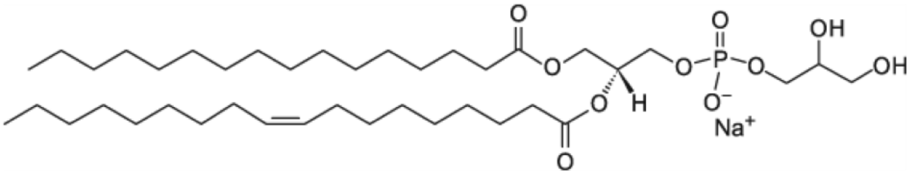

1-palmitoyl-2-oleoyl-sn-glycero-3-phospho-(1'-rac-glycerol) (sodium salt) (**POPG**)

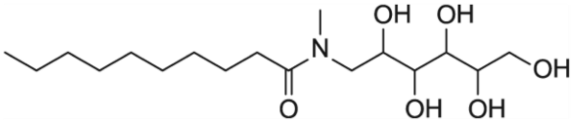

N-decanoyl-N-methylglucamine (**MEGA-10**)

Figure S1. The chemical structure of components used to generate liposomes from dilution.

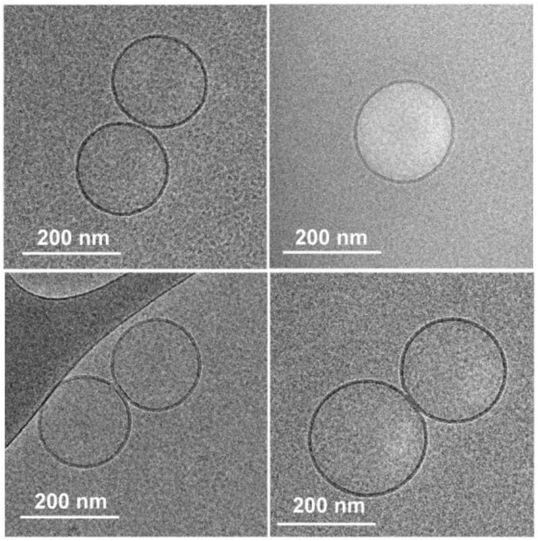

Figure S2. Representative cryo-TEM micrographs of 200 nm liposomes formed in region iii.

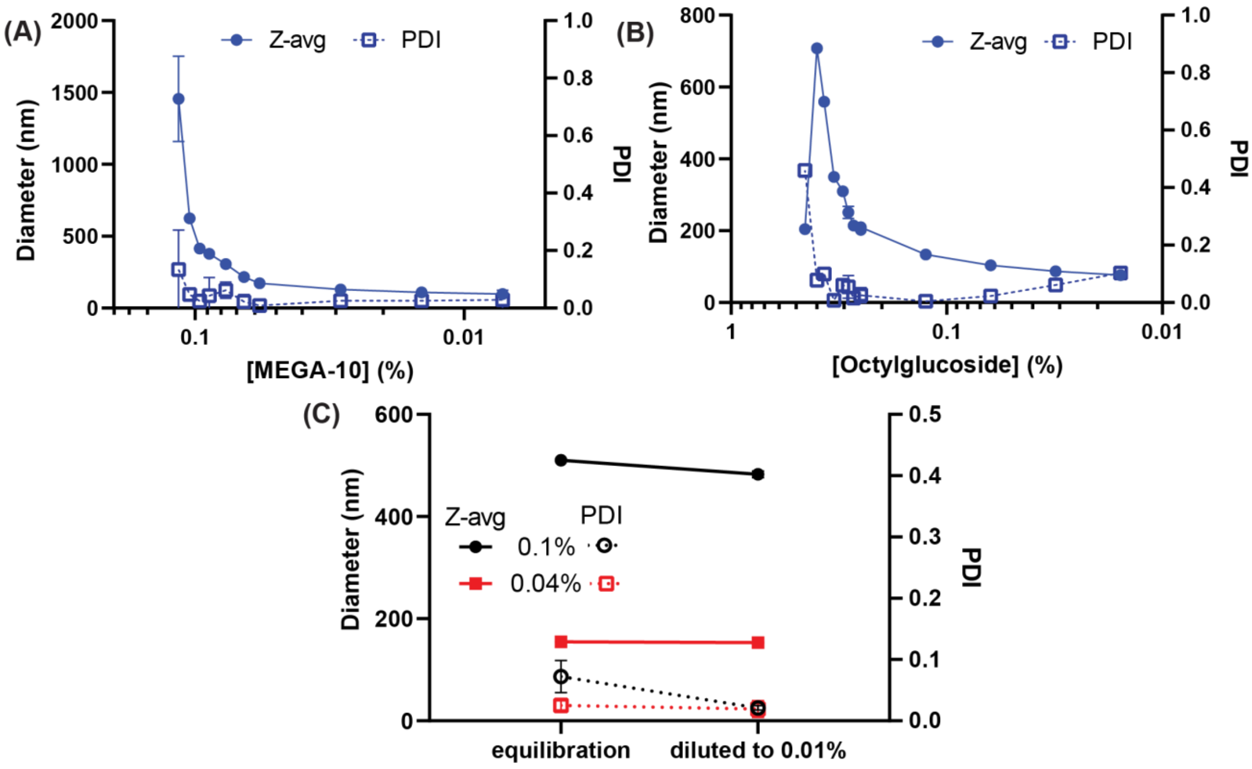

Figure S3. Detergent concentration after dilution controls the equilibrium size of liposomes. **(A)** DLS intensity-weighted size (Z-avg) and PDI after overnight incubation of a 10 mg/mL of 6:3:1 molar mixture of DSPC:cholesterol:POPG in 10% MEGA-10 diluted to various final detergent concentrations. (**B**) DLS intensity-weighted size (Z-avg) and PDI after overnight incubation of a 20 mg/mL of 6:3:1 molar mixture of DSPC:cholesterol:POPG in 10% octylglucoside diluted to various final detergent concentrations. **(C)** Effect of dilution samples equilibrated at 0.1% or 0.04% MEGA-10 overnight to 0.01% MEGA-10 on particle size (Z-avg) and PDI; samples were allowed to equilibrate at 0.01% overnight.

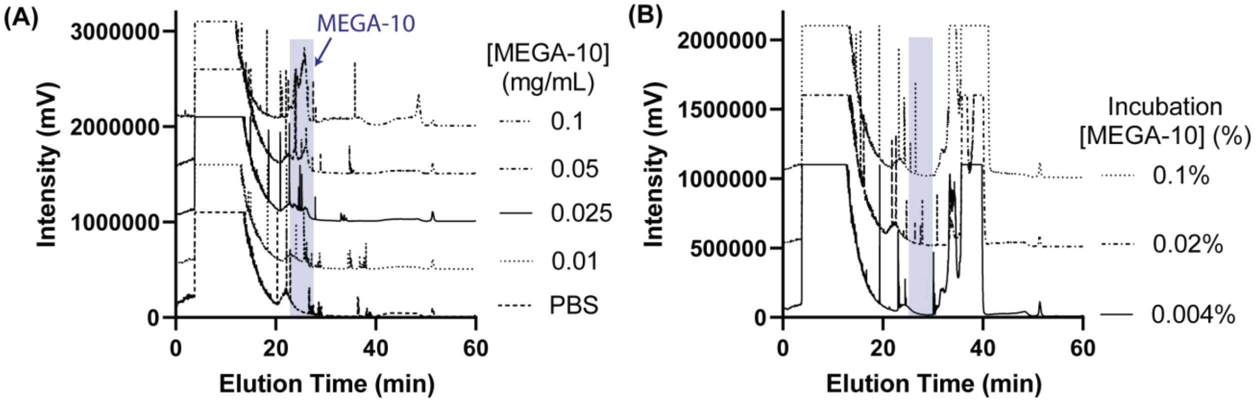

Figure S4. TFF of assembled lipid vesicles enables efficient removal of MEGA-10. **(A)** ELSD chromatogram of MEGA-10 at increasing concentrations. **(B)** ELSD of final purified particles incubated at either 0.1%, 0.02%, or 0.004% MEGA-10. Shaded regions indicate MEGA-10 elution times.

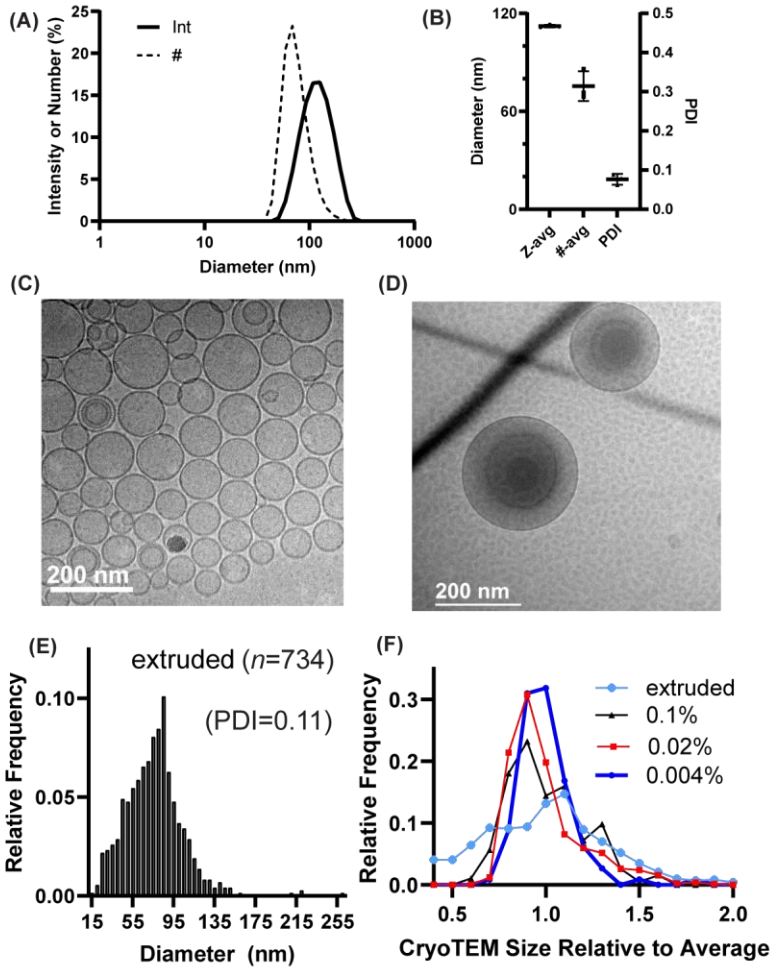

Figure S5. Characteristics of liposomes generated from lipid film hydration followed by extrusion with a 50 nm pore sized membrane. **(A)** DLS intensity-based and number-based size distribution. **(B)** Z-avg, number-average, and PDI. **(C)** Representative cryoTEM micrograph from liposomes generated via thin film hydration followed by extrusion on a 50 nm pore-sized membrane. **(D)** CryoTEM micrograph of extruded liposomes showing large (>50 nm) vesicles. **(E)** CryoTEM micrograph analysis of particle size from the extruded sample. Parenthesis indicates the total number of particles quantified and PDI based on the measured particle sizes from cryoTEM. **(F)** Normalized histograms of samples generated from detergent dilution into 0.1%, 0.02%, and

0.004% MEGA-10 then purified via TFF compared to liposomes generated via thin-film hydration followed by extrusion through 50 nm pore membrane.

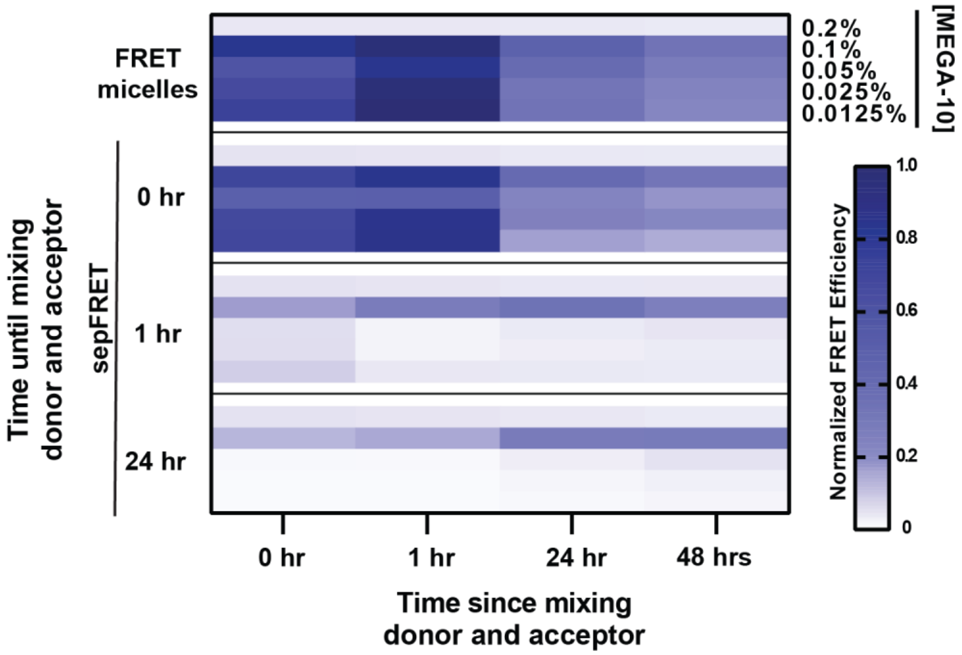

Figure S6. Analysis of lipid exchange via FRET reveals that assembled liposomes in region iv are stable whereas particles in region iii have high rates of lipid exchange even after no change in particle size. Normalized FRET efficiency from samples containing 1mol% of dye diluted with PBS to various concentrations of MEGA-10 and mixed after set incubation periods.

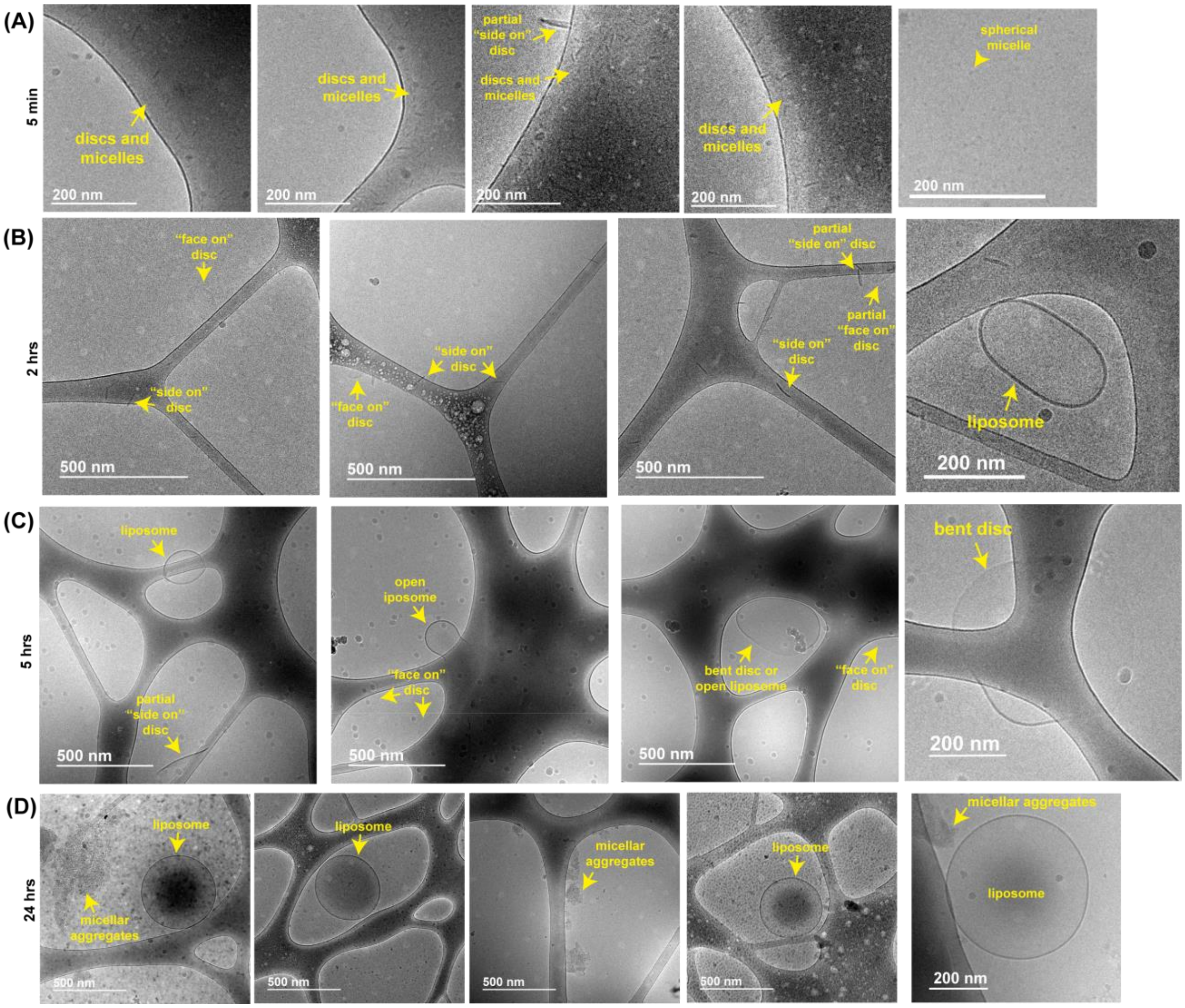

Figure S7. Assessment of liposome assembly via cryo-TEM. **(A-D)** Cryo-TEM micrographs of 10 mg/mL 6:3:1 DSPC:Chol:POPG sample in 10% MEGA-10 rapidly diluted to 0.1% MEGA-10 with 10 mM HEPES 150 mM NaCl and frozen 5 minutes, 2 hours, 5 hours and 24 hours after dilution, respectively.

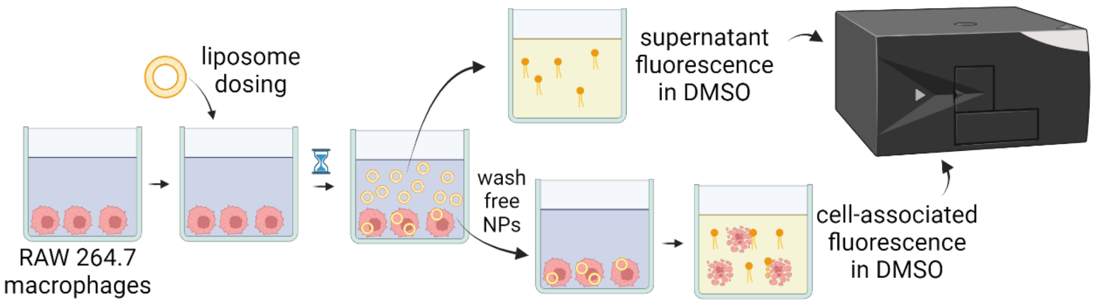

Figure S8. Diagram of the experimental protocol to determine the percentage of liposome fluorescence associated with macrophages.
